# Risk assessment of the onset of Osgood–Schlatter disease using kinetic analysis of various motions in sports

**DOI:** 10.1371/journal.pone.0190503

**Published:** 2018-01-08

**Authors:** Gento Itoh, Hideyuki Ishii, Haruyasu Kato, Yasuharu Nagano, Hiroteru Hayashi, Hiroki Funasaki

**Affiliations:** 1 Graduate School of Community and Human Services, Rikkyo University, Saitama, Japan; 2 Department of Sport and Wellness, Rikkyo University, Saitama, Japan; 3 Department of Sports Wellness Sciences, Japan Women's College of Physical Education, Tokyo, Japan; 4 Department of Sports and Wellness Clinic, Jikei University School of Medicine, Tokyo, Japan; Tokai University, JAPAN

## Abstract

**Background:**

Some studies have listed motions that may cause Osgood-Schlatter disease, but none have quantitatively assessed the load on the tibial tubercle by such motions.

**Purposes:**

To quantitatively identify the load on the tibial tubercle through a biomechanical approach using various motions that may cause Osgood-Schlatter disease, and to compare the load between different motions.

**Methods:**

Eight healthy male subjects were included. They conducted 4 types of kicks with a soccer ball, 2 types of runs, 2 types of squats, 2 types of jump landings, 2 types of stops, 1 type of turn, and 1 type of cutting motion. The angular impulse was calculated for knee extension moments ≥1.0 Nm/kg, ≥1.5 Nm/kg, ≥2.0 Nm/kg, and ≥2.5 Nm/kg. After analysis of variance, the post-hoc test was used to perform pairwise comparisons between all groups.

**Results/Conclusions:**

The motion with the highest mean angular impulse of knee extension moment ≥1.0 Nm/kg was the single-leg landing after a jump, and that with the second highest mean was the cutting motion. At ≥1.5 Nm/kg, ≥2.0 Nm/kg, and ≥2.5 Nm/kg, the cutting motion was the highest, followed by the jump with a single-leg landing. They have a large load, and are associated with a higher risk of developing Osgood-Schlatter disease. The mean angular impulse of the 2 types of runs was small at all the indicators.

**Clinical relevance:**

Motions with a high risk of developing Osgood-Schlatter disease and low-risk motions can be assessed in further detail if future studies can quantify the load and number of repetitions that may cause Osgood-Schlatter disease while considering age and the development stage. Scheduled training regimens that balance load on the tibial tubercle with low-load motions after a training day of many load-intensive motions may prevent athletes from developing Osgood-Schlatter disease and increase their participation in sports.

## Introduction

Osgood–Schlatter disease was discovered as an injury in which the tibial tubercle is strained during adolescence [1, 2]. Ehrenborg et al. found that Osgood–Schlatter disease is likely to occur during the apophyseal stage, where an ossified center develops in the ligulate part of the tibial tubercle cartilage [3]. Ogden et al. noted that Osgood–Schlatter disease is caused by an inability of the secondary ossification center to withstand the repeated tension created by the quadriceps and patellar tendon [4]. Rosenberg et al. suggested that an inflammation in tibial tubercles and soft tissue is more likely the cause of Osgood–Schlatter disease [5]. These past studies suggest that Osgood–Schlatter disease is caused by inflammation from repeated friction of the quadriceps on the epiphyseal cartilage in the tibial tubercle during a kinetically weak apophyseal stage. Osgood-Schlatter disease is associated with more in sports that involve jumping, squating, kicking, and running [6, 7]. Specifically problematic sports include basketball, volleyball, and soccer [8]. Generally, the disease develops in many children that undergo rapid growth during puberty [9, 10], which are boys aged 10–15 and girls aged 8–14 [11].

Major methods of treatment are icing, massage, limiting exercise, painkillers, stretching the quadriceps and hamstrings, and fixation of the knee joints [6, 12–17]. One study reported that athletes who suffer from Osgood–Schlatter disease stop training for an average of 3.2 months and limit training for an average of 7.3 months [9].

Osgood–Schlatter disease is an injury in which inflammation occurs in the epiphyseal cartilage of the tibial tubercle by repeated traction [3–5]. Young athletes are thought to suffer from injuries such as Osgood–Schlatter disease through overuse of the body when it cannot cope with the stress incurred on the tibial tubercle while it is still mechanically vulnerable [9, 18]. Some studies have listed motions that may cause the disease, but none have quantitatively assessed the load on the tibial tubercle by such motions. To prevent from the onset and aggravation of Osgood–Schlatter disease, it may be effective if coaches develop a training regimen that controls the load on the tibial tubercle. To do so, the load needs to be identified while they are in motion. The objective of this study is to quantitatively identify the load on the tibial tubercle through a biomechanical approach in various motions that may cause Osgood–Schlatter disease and compare the load between different motions. Many of the motions analyzed by this study occur in many sports, and the onset and aggravation of Osgood–Schlatter disease may be prevented by prohibiting prolonged load-intensive training across various sports. Some motions cited as developing Osgood-Schlatter disease by many researches may be identified that have large loads or small loads in this study.

## Materials and methods

This study investigated the load on the tibial tubercle in the most common motions in sports such as soccer and basketball. Considering the proficiency required to perform a kicking motion in soccer, 8 healthy males, with an average of 10 years of soccer experience (age: 22.4±0.7, height: 1.713±0.053 m, weight: 63.6±5.1 kg; right-footed) were employed as test subjects. Adult males were chosen as test subjects considering the risks involved in testing with boys, since boys aged 12 to 15 are likely to develop Osgood–Schlatter disease [19], and their bones are kinetically weak, having a significant amount of cartilage tissues. Employing adults as test subjects should not be a problem for this study, since its objective is to compare the load on the tibial tubercle among 14 types of motion relative to each other, rather than discussing the absolute values of the load.

This study conducted tests under the approval of the Ethics and Safety Committee for research and experiments in life sciences at Rikkyo University. The objective, procedures, measured items, and risks were explained to every test subject in advance, and they were asked to sign an agreement form to participate in the test.

Rigorous running, jumping, stops, sharp changes in direction and squats are thought of as the cause of Osgood-Schlatter disease [20–22]. The test subjects conducted 4 types of kicks, 2 types of runs, 2 types of squats, 2 types of jumps, 1 type of turn, 1 type of cutting motion, and 2 types of stops, for a total of 14 motions. A series of kicks were chosen as testing motions since they are essential in soccer, which is thought to be prone to cause Osgood–Schlatter disease. Running, jumping, turning, cutting, and stopping were also chosen as they are basic motions in various sports, including soccer and basketball. The 4 types of kicking motions were all instep kicks used in soccer, with the left foot acting as the pivot foot. The easiest type of kick for the test subjects was called a "Kick Normal" (S1 Movie), a kick where the knee joint is flexed more than the "Kick Normal" was called a "Kick Flexion" (S2 Movie), a kick where the knee joint of the pivot foot is extended more than the "Kick Normal" was called a "Kick Extension" (S3 Movie), and a kick while running forward was called a "Kick Run" (S4 Movie). Runs were defined as a run at a jogging speed ("Run Slow") and a full-speed run ("Run Fast") (S5 and S6 Movies). Squats were defined as a squat in which movement takes place in the hip joint with a fixed knee position ("Squat Hip") and a squat in which movement takes place in the knee joints ("Squat Knee") (S7 and S8 Movies). Jumps were defined as a vertical jump in which test subjects land with both legs ("Jump Both Legs Landing") and a vertical jump in which test subjects land with one leg ("Jump Single Leg Landing") (S9 and S10 Movies). A "Turn" involved a 180 degree turn from a full-speed run (S11 Movie). A "Cutting" motion involved a change in direction to the front right while running full-speed (S12 Movie). Stops were defined in which test subjects stop abruptly from a full-speed run ("Approach Stop") and a stop in which a test subject stops from a full-speed run and starts running backwards ("Approach Stop & Back") (S13 and S14 Movies). Test subjects were instructed to land on the force plate with their left leg in all of their test motions.

A spherical reflection marker with a 9.5-mm diameter was placed on 18 points across the body of the test subjects (anterior superior iliac spine, posterior superior iliac spine, greater trochanter, lateral femoral condoyle, medial femoral condoyle, lateral malleolus, medial malleolus, and calcaneal tuberosity on both sides of the body, toe on the right foot, and left foot second metatarsal head) (S1 Fig). Each motion was captured with 8 optical motion capturing cameras (Vicon, UK) at 200 Hz and the positional coordinates of each marker were also obtained. A force plate (Kistler, Switzerland) was also used to measure the ground reaction force on the left foot at 1000 Hz.

The hip joint center was estimated from the positions of both greater trochanters [23]. The center of mass of the lower limb was estimated from the position of the center of the knee joint (the midpoint between femur lateral condoyle and medial femoral condoyle) and the center of the ankle joint (the midpoint between foot malleolus and lateral malleolus), which was based on the body inertia coefficient [24]. The center of mass of the foot was obtained from the positions of the center of the ankle joint and second metatarsal femoral head [25]. Positional coordinates and ground reaction force data was smoothed by a fourth order Butterworth digital low pass filter where the cutoff frequency was determined by residual analysis [26].

A moving coordinate system was defined for femur, lower limb, and foot based on the joint coordinate calculation method by Grood and Suntay [27]. Knee extension and flexion angles were calculated using the Euler angle from the moving coordinates.

The lower left limb was modeled as a rigid link segment model consisting of a femur, lower limb, and a foot. Mass and inertial moment of each segment was calculated using a body moment of inertia coefficient [24], and the knee extension moment was calculated by solving the Newton-Euler law of motion by inverse dynamics. The knee extension moment was standardized by dividing the calculated value of the joint moment by the weight in order to remove the impact of the weight differences of the test subjects. Osgood–Schlatter disease is thought to be caused by a repeated exertion of traction force by extending the knee joint and knee flexion to the tibial tubercle, to which the still maturing patellar tendon is attached [28]. For this reason, the present study treated the knee extension moment determined by the tension in the patellar tendon as a load and compared it with loads involved in other test motions.

The range of calculation for knee extension moments of the kicks (4 types) was defined as the moment in which the left foot landed on the force plate, to when the right foot impacted the ball. The range of calculation for the knee extension moment of runs (2 types), "Turn", "Cutting", and "Approach Stop & Back" was defined as the moment in which the left foot landed on the force plate to the moment it left the plate, and the knee extension moment of "Approach Stop" was defined as the moment the left foot landed on the force plate to the moment the right foot landed on the ground. The range of calculation for the knee extension moment of jumps (2 types) was defined as the moment the left foot landed on the force plate to the moment in which the body stopped, and the range of calculation for the knee extension moment of squats (2 types) was defined as the moment the motion started to the moment the motion stopped.

The peak value was calculated for the knee extension moment of each motion and was defined as the value at which the knee extension moment is the largest within the range of calculations. The angular impulse was also calculated by integrating the knee extension moment value with respect to time. The angular impulse reflects the size of the knee extension moment including the peak value and time of action. However, the angular impulse was calculated for knee extension moments greater than or equal to 1.0 Nm/kg. This constraint ensured that the values reflect the degree of risk for Osgood–Schlatter disease by assuring that the indicator value is not overestimated, even if small moments such as standing or walking take place for an extended period of time. However, the specific size of the load that causes Osgood–Schlatter disease is not known, and there may be a more appropriate indicator. Given that there is a 5-year age gap when different sexes are most likely to develop Osgood–Schlatter disease (10–15 for boys, 8–14 for girls [11]), there is a possibility that the load that is considered needs to be differentiated depending on the age and the stage of development. Therefore, angular impulse was also calculated for the knee extension moment greater than or equal to 1.5 Nm/kg, greater than or equal to 2.0 Nm/kg, and greater than or equal to 2.5 Nm/kg. Mean angular impulse of the knee extension moment for all test subjects was also calculated for each motion and compared with each other.

Statistical analysis software SPSS (IBM, ver. 22) was used to test the different motions in terms of the peak knee extension moment and mean angular impulse. After using the one-way analysis of variance (ANOVA), multiple comparisons (post-hoc test) were performed by the Tukey method, which gave pairwise comparisons between all groups. The velocity of the horizontal component of both the left and right anterior superior iliac spine was calculated as the running speed at the moment immediately before the left foot of the test subject touched the force plate, for 10 types of motion, including 4 types of kicks, 2 types of stops, 1 type of cutting motion, 1 type of turn, and 2 types of runs. The difference between types of motion were tested for an average running speed of 10 types of motion using the same method as with the peak knee extension moment and angular impulse. The difference between the highest reaching point of the left and right anterior superior iliac spine during a jump and the height while standing still were calculated as the jumping height for both types of jumping motions. The difference between motions was also tested for the mean jumping height using a t-test. The significance level was set at 5% for all tests.

## Results

**Fig 1:**
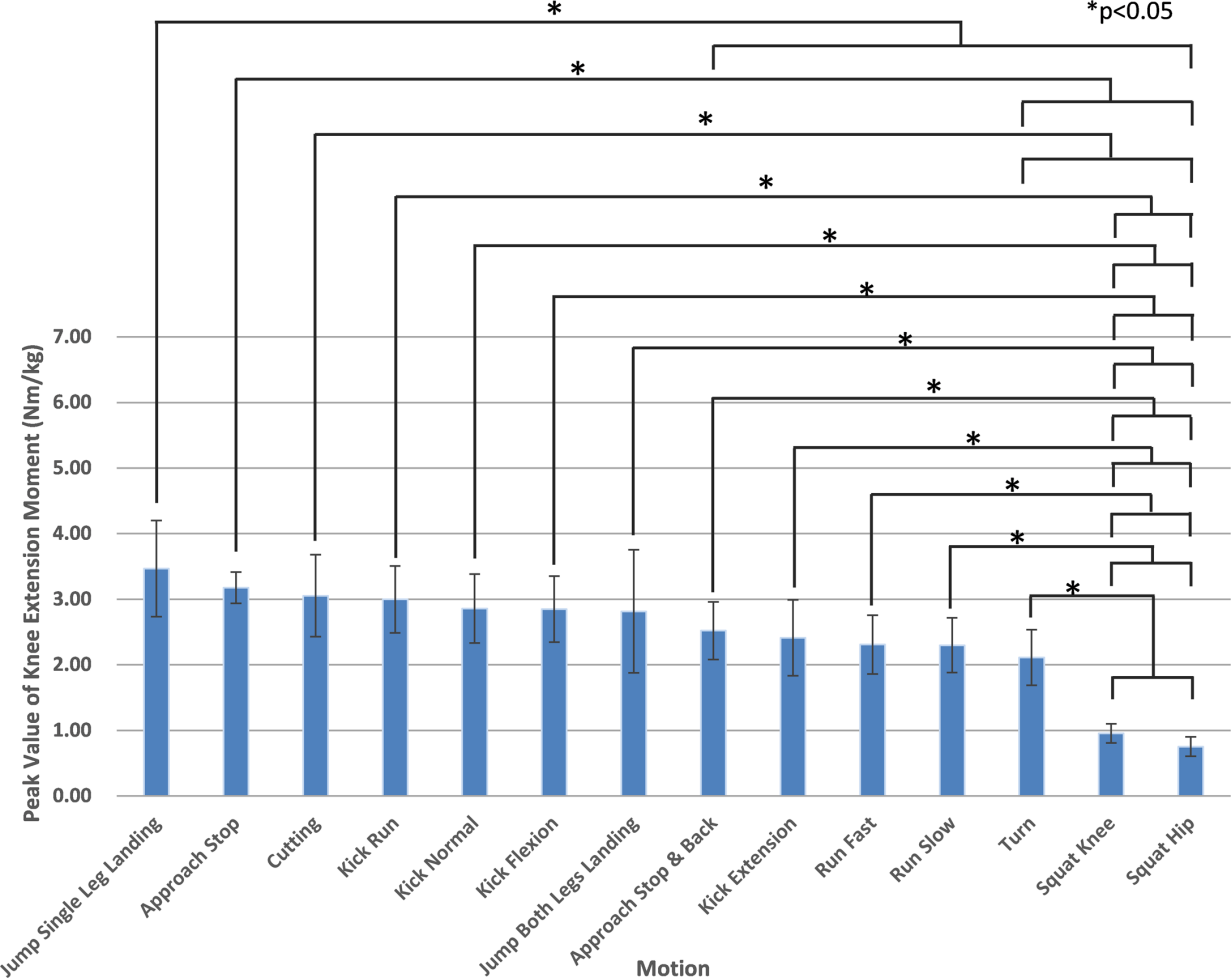
Comparison between motions by test subject mean of peak knee extension moment. Values are shown in descending order from left to right. Multiple comparisons were performed after ANOVA to show statistically significant differences between the motions. *P<0.05.

Table 1 shows the peak knee extension moment per test subject and the average for each motion. Fig 1 shows the mean values in Table 1 ranked from largest to smallest. The motion with the highest average was for the "Jump Single Leg Landing" (3.468 Nm/kg). The motion with the second highest mean was "Approach Stop" (3.177 Nm/kg), followed by "Cutting" (3.054 Nm/kg). "Squat Hip" (0.754 Nm/kg) was the motion with the lowest mean.

**Table 1:** Peak knee extension moment per test subject for each motion (Nm/kg).

		Subject ID								Mean	SD
		A	B	C	D	E	F	G	H		
**Kick**	**Normal**	2.85	3.20	2.30	3.91	2.58	3.00	2.68	2.35	2.858	0.525
**Kick**	**Flexion**	3.10	2.51	2.50	3.81	2.49	2.48	2.58	3.32	2.849	0.505
**Kick**	**Extension**	2.66	2.04	2.00	3.70	1.93	2.16	2.31	2.49	2.411	0.578
**Kick**	**Run**	3.10	2.28	2.44	3.40	3.60	3.35	2.52	3.30	2.998	0.509
**Approach**	**Stop**	3.46	3.29	2.78	3.02	3.08	3.40	3.37	3.02	3.177	0.238
**Approach**	**Stop & Back**	3.03	1.99	1.98	2.96	2.30	2.29	2.99	2.64	2.521	0.440
**Cutting**		3.72	2.10	2.29	3.74	3.01	3.55	2.82	3.20	3.054	0.625
**Turn**		2.06	2.07	1.78	1.76	1.62	2.12	2.69	2.78	2.110	0.424
**Jump**	**Both Legs Landing**	3.79	1.76	2.47	4.19	1.78	3.61	2.72	2.19	2.816	0.938
**Jump**	**Single Leg Landing**	3.68	2.53	2.56	4.83	3.35	3.58	3.38	3.82	3.468	0.735
**Run**	**Slow**	2.92	2.57	1.93	2.17	2.34	2.55	1.56	2.35	2.299	0.418
**Run**	**Fast**	2.34	1.93	1.85	2.52	2.12	2.35	2.09	3.27	2.310	0.448
**Squat**	**Knee**	0.97	1.14	0.71	0.89	0.85	1.14	0.92	1.02	0.955	0.147
**Squat**	**Hip**	0.49	0.84	0.60	0.95	0.75	0.73	0.84	0.83	0.754	0.147

**Fig 2:**
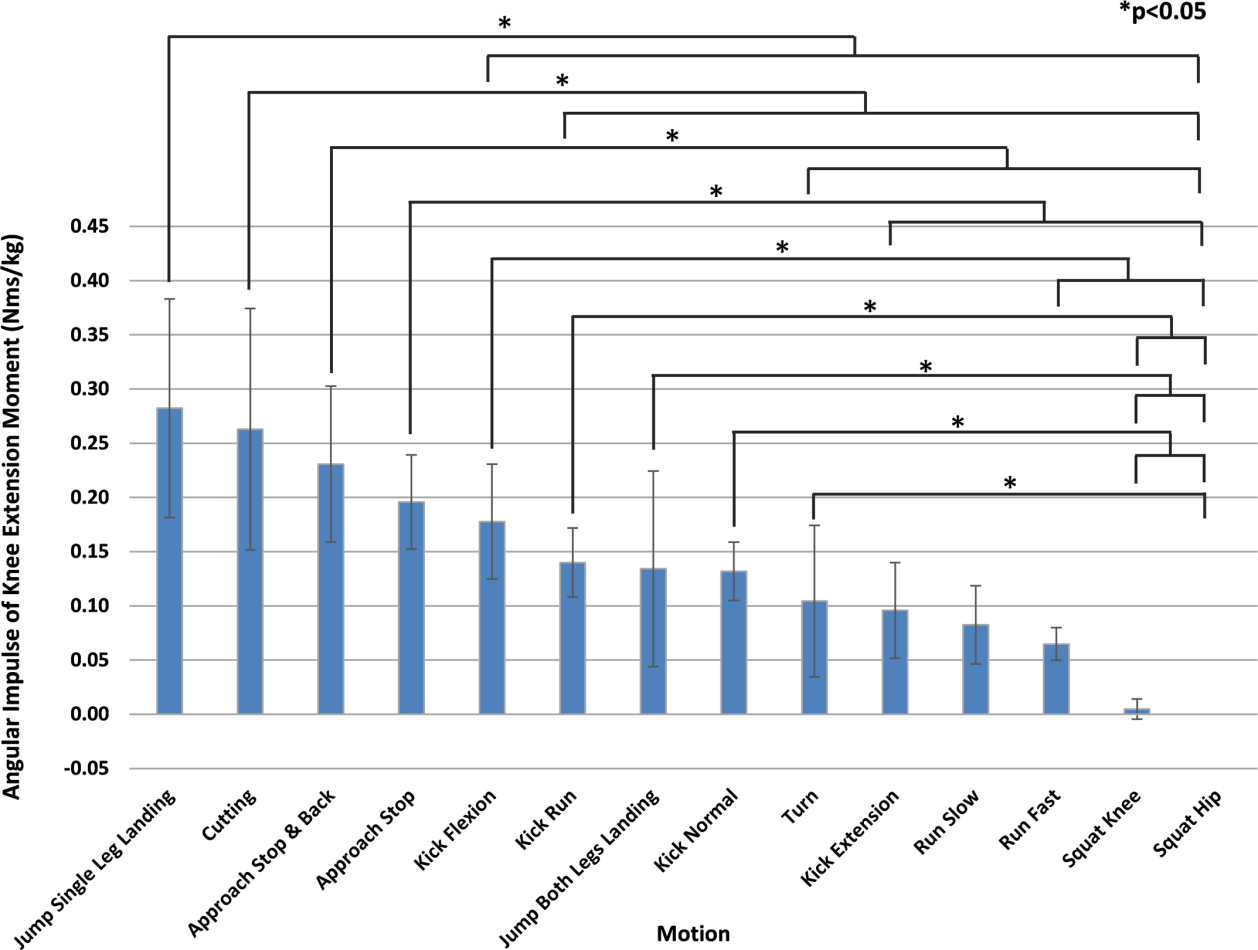
Motion comparison by average angular impulse of the knee extension moment (1.0 Nm/kg or greater). Mean values are shown in descending order from left to right. "Jump Single Leg Landing" was significantly greater than "Kick Flexion" with other motions ranked below that. Multiple comparisons were performed after ANOVA to show statistically significant differences between motions. *P<0.05.

Table 2 shows the angular impulse of the knee extension moment greater than or equal to 1.0 Nm/kg per test subject for each motion, as well as their mean and standard deviation. Fig 2 ranks the mean shown in Table 2 in descending order. The motion with the highest mean angular impulse of the knee extension moment was "Jump Single Leg Landing" (0.282 Nms/kg). The motion with the second highest mean was "Cutting" (0.263 Nms/kg). This was followed by "Approach Stop & Back" (0.231 Nms/kg). "Squat Hip" (0.000 Nms/kg) was the motion the lowest mean, which never exceeded 1.0 Nm/kg. The angular impulse of the knee extension moment of "Jump Single Leg Landing" was significantly greater than "Kick Flexion" with other motions ranked below that. The angular impulse of "Cutting" was significantly greater compared to "Kick Run" and other motions ranked below that.

**Table 2:** Angular impulse of the knee extension moment per test subject for each motion. Note that the angular impulse in this study is the integral of the moments that are greater than or equal to 1.0 Nm/kg (Nms/kg).

		Subject ID								Mean	SD
		A	B	C	D	E	F	G	H		
**Kick**	**Normal**	0.15	0.14	0.08	0.15	0.13	0.17	0.13	0.11	0.132	0.027
**Kick**	**Flexion**	0.23	0.16	0.13	0.24	0.21	0.10	0.15	0.22	0.178	0.053
**Kick**	**Extension**	0.16	0.05	0.04	0.13	0.08	0.06	0.09	0.15	0.096	0.044
**Kick**	**Run**	0.17	0.11	0.10	0.15	0.18	0.18	0.11	0.13	0.140	0.032
**Approach**	**Stop**	0.25	0.15	0.24	0.17	0.23	0.15	0.21	0.15	0.196	0.043
**Approach**	**Stop & Back**	0.28	0.15	0.14	0.33	0.18	0.31	0.23	0.21	0.231	0.072
**Cutting**		0.40	0.07	0.22	0.38	0.32	0.30	0.16	0.24	0.263	0.111
**Turn**		0.05	0.05	0.06	0.09	0.06	0.12	0.14	0.26	0.104	0.070
**Jump**	**Both Legs Landing**	0.26	0.06	0.03	0.18	0.08	0.27	0.14	0.06	0.134	0.090
**Jump**	**Single Leg Landing**	0.35	0.29	0.07	0.36	0.23	0.35	0.24	0.36	0.282	0.101
**Run**	**Slow**	0.12	0.12	0.04	0.06	0.09	0.13	0.04	0.07	0.082	0.036
**Run**	**Fast**	0.08	0.05	0.05	0.08	0.05	0.06	0.07	0.09	0.065	0.015
**Squat**	**Knee**	0.00	0.01	0.00	0.00	0.00	0.02	0.00	0.00	0.005	0.009
**Squat**	**Hip**	0.00	0.00	0.00	0.00	0.00	0.00	0.00	0.00	0.000	0.000

**Fig 3:**
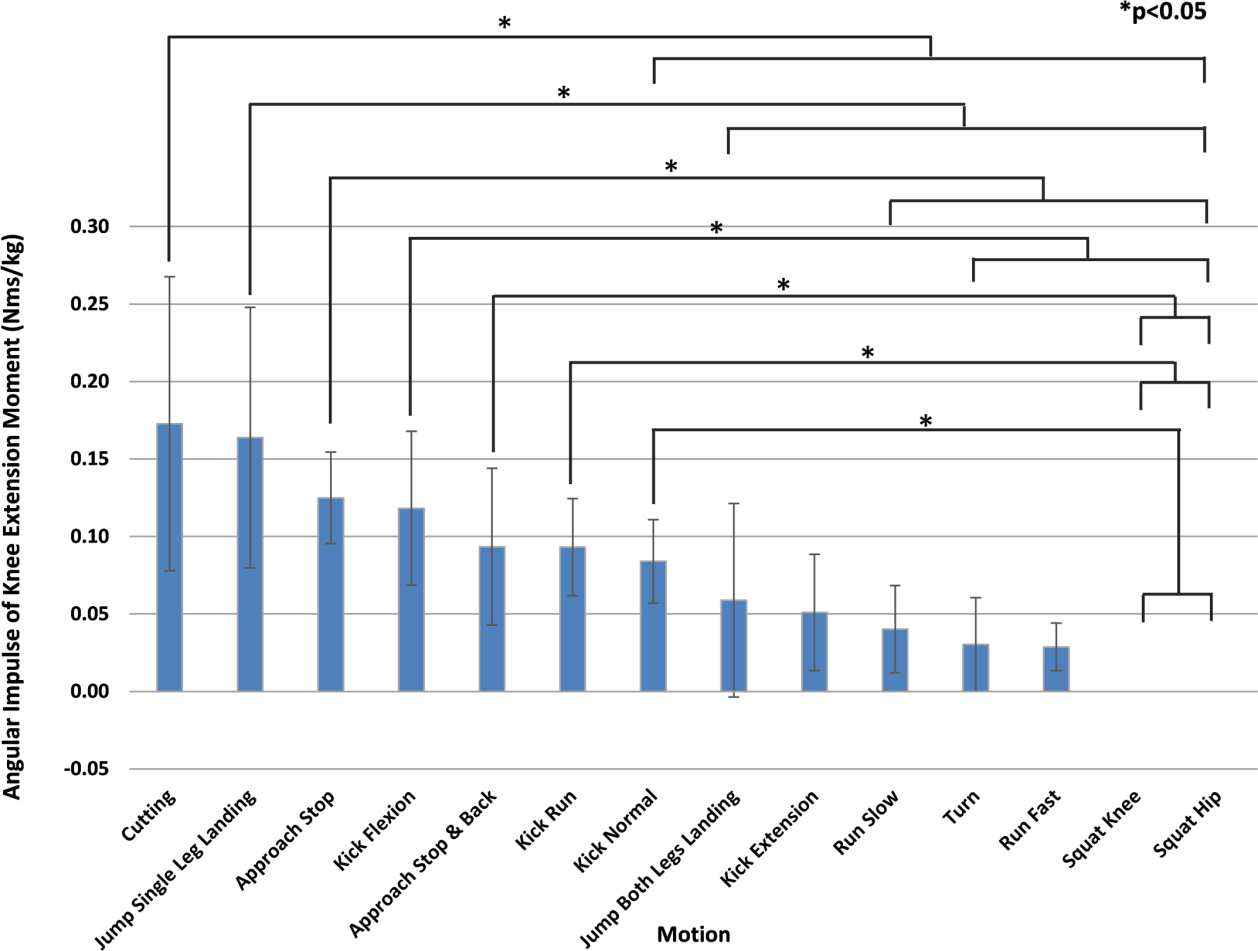
Motion comparison by average angular impulse of the knee extension moment (1.5 Nm/kg or greater). Mean values are shown in descending order from left to right. "Cutting" was significantly greater than 8 types of motion, and "Jump Single Leg Landing" was significantly greater than 7 types of motion. Multiple comparisons were performed after ANOVA to show statistically significant differences between motions. *P<0.05.

Fig 3 ranks the mean angular impulse of the knee extension moment greater than or equal to 1.5 Nm/kg in descending order. The motion with the highest mean angular impulse was "Cutting" (0.173 Nms/kg), followed by "Jump Single Leg Landing" (0.164 Nms/kg). The mean angular impulse for "Squat Knee" and "Squat Hip" were 0.000 Nms/kg and their knee extension moments never exceeded 1.5 Nm/kg. The angular impulse of "Cutting" for moment greater than or equal to 1.5 Nm/kg was significantly greater than 8 types of motion, and "Jump Single Leg Landing" was significantly greater than 7 types of motion including "Jump Both Legs Landing".

**Fig 4:**
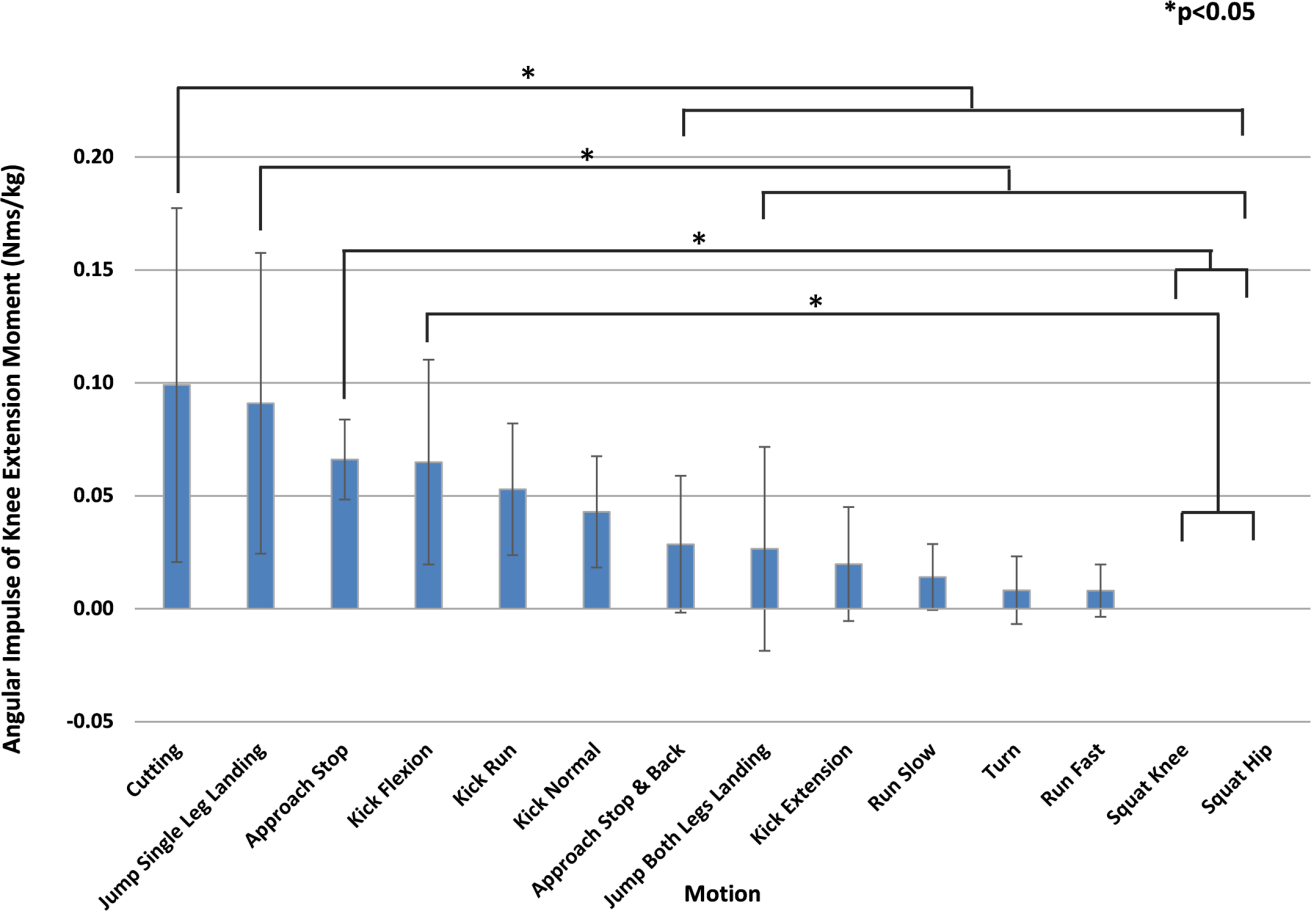
Motion comparison by average angular impulse of the knee extension moment (2.0 Nm/kg or greater). Mean values are shown in descending order from left to right. "Cutting" was significantly greater than 8 types of motion. Multiple comparisons were performed after ANOVA to show statistically significant differences between motions. *P<0.05.

Fig 4 ranks the mean angular impulse of the knee extension moment greater than or equal to 2.0 Nm/kg in descending order. The motion with the highest mean angular impulse greater than or equal to 2.0 Nm/kg was "cutting" (0.099 Nms/kg), followed by "jump single leg landing" (0.091 Nms/kg). "Cutting", whose angular impulse was the largest for moments greater than or equal to 2.0 Nm/kg, was significantly greater than 8 types of motion including "Approach Stop & Back", and the second largest "Jump Single Leg Landing" was significantly higher than 7 types of motion.

**Fig 5:**
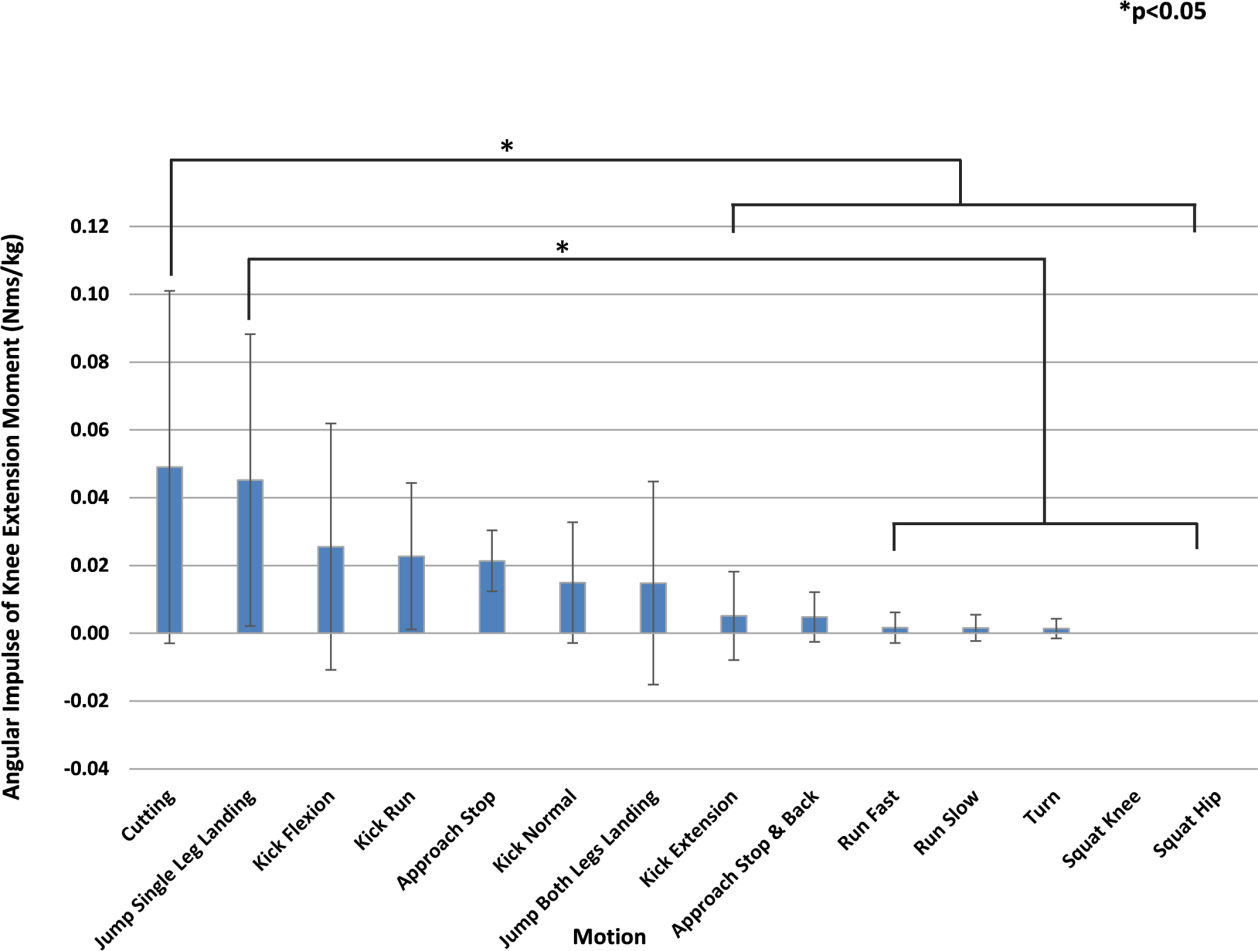
Motion comparison by average angular impulse of the knee extension moment (2.5 Nm/kg or greater). Mean values are shown in descending order from left to right. The angular impulse of "Cutting" was significantly greater than 7 types of motion. Multiple comparisons were performed after ANOVA to show statistically significant differences between motions. *P<0.05.

Fig 5 ranks the angular impulse of the knee extension moment greater than or equal to 2.5 Nm/kg for each motion in descending order. "Cutting" (0.049 Nms/kg) was the largest, and "Jump Single Leg Landing" (0.045 Nms/kg) ranked second. The angular impulse of "Cutting" was significantly greater than 7 types of motion. "Jump Single Leg Landing" was significantly greater than 5 types of motion.

## Discussion

This study aims to quantitatively identify the knee extension moment (i.e., the load on the tibial tubercle) in various motions that may cause Osgood-Schlatter disease and compare the load between different motions. The findings of this study can contribute to protecting many young athletes from the disease and also preventing a decrease of their participation in sports.

It is commonly believed that excessive running leads to the development of Osgood–Schlatter disease [20], but the angular impulse of the knee extension moment greater than or equal to 1.0 Nm/kg measured for running motions were small in this study. Running cannot be viewed as a motion that exerts a large load because there was no statistically significant difference with "Squat Hip" whose angular impulse was the smallest. While there was no statistically significant difference, the peak knee extension moment was greater for "Run Fast" while the angular impulse was greater for "Run Slow", which was caused largely by a longer period of contact between the foot and the ground due to a slower running speed (“Run Slow”: 2.81±0.28 m/s, “Run Fast”: 5.14±0.23 m/s, p<0.05). As noted before, Osgood–Schlatter disease is likely to develop and aggravate in sports that involve a significant level of running. Running is a motion that occurs frequently in many sports, and excessive running may cause and aggravate Osgood–Schlatter disease to develop regardless of the speed. However, the results from this study suggested that running cannot be viewed as a motion that exerts a large load.

Repeated jumps are also believed to cause Osgood–Schlatter disease [20]. Calculated angular impulse of knee extension moment at 1.0 Nm/kg or greater showed that single-legged landing ("Jump Single Leg Landing") was the motion with the largest load among the 14 types of motion assessed in this study. "Jump Single Leg Landing" was significantly greater than "Jump Both Legs Landing" in terms of both peak moment and angular impulse, which suggests that landing with one leg exerts a greater load than landing with both legs. No statistically significant difference was observed in jumping height between the two motions ("Jump Single Leg Landing": 0.40±0.04 m, "Jump Both Legs Landing": 0.45±0.08 m), which shows the jump height was not the reason for a greater load in single legged landing. The findings above suggested that the loan on the tibial tubercle could be mitigated by landing with both legs rather than one when jumping in training sessions.

Stops are also thought to provide sustained stress to the knee extension mechanism [21]. Angular impulse of knee extension moment at 1.0 Nm/kg or greater of "Approach Stop" was the fourth largest and significantly greater than 5 other types of motion ("Turn" to "Squat Hip"). "Kick Flexion", the fifth largest, was significantly different from only 3 types of motion ("Run Fast" and both squats), thus "Approach Stop" was deemed to have a greater load than the types of motion ranked below, including "Kick Flexion". However, the load by "Approach Stop" was still smaller than the types of motion ranked higher than "Cutting", since the number of types of motion with which it had a significant difference were fewer than "Cutting" and "Jump Single Leg Landing". The angular impulse of "Approach Stop & Back" was the third largest and significantly greater than 6 other types of motion, thus it was deemed to be a load-intensive motion. Similarly, "Approach Stop & Back" was deemed to have a larger load than "Approach Stop" since the latter was significantly greater than 5 types of motion. The load by "Approach Stop & Back" was not on the same level as "Jump Single Leg Landing" or "Cutting", as it had fewer statistically significant differences from the other types of motion. While there were no significant differences between the 2 types of stop motions, the angular impulse was greater for "Approach Stop & Back" than for "Approach Stop". The running speed immediately before the left foot landed on the force plate was 2.48±0.38 m/s for "Approach Stop & Back" and 3.11±0.29 m/s for "Approach Stop", and no statistically significant difference was observed. Therefore, running speed was not the reason why the angular impulse for "Approach Stop & Back" was greater than it was in "Approach Stop". As opposed to "Approach Stop", in which the test subjects simply stop after running full-speed, test subjects had to kick the ground and apply a significant reaction force to run back after stopping in "Approach Stop & Back", which caused the angular impulse to be large. While stop motions were suspected to have a large load based on the above findings, the results showed that stopping exerts less load than single-legged landing after a jump or a sharp change in direction.

Sharp changes in direction are also thought to cause Osgood–Schlatter disease [21]. The angular impulse of knee extension moment at 1.0 Nm/kg or greater of "Cutting" was the second largest and significantly greater than 9 types of motion ("Kick Run" to "Squat Hip"). Since the third-ranked "Approach Stop & Back" was significantly greater than 6 types of motion ("Turn" to "Squat Hip") while "Jump Single Leg Landing" was significantly greater than 10 types of motion, "Cutting" was a motion that exerted less load than "Jump Single Leg Landing", and a greater load than motions ranked below it, including, "Approach Stop & Back". In light of the above, sharp changes in direction ("Cutting") were deemed to be load-intensive on the tibial tubercle, albeit with less load compared to single-legged landing after a jump.

Since the load size that develops and aggravates Osgood–Schlatter disease is not known to this day, there may be a more appropriate indicator than moments greater than 1.0 Nm/kg. There is also a possibility that appropriate indicators may vary by age and stage of development of the child. Therefore, in addition to the knee extension moment at 1.0 Nm/kg or greater as an indicator, this study also chose other indicators such as the moment at 1.5 Nm/kg or greater, 2.0 Nm/kg or greater, and 2.5 Nm/kg or greater, and calculated the angular impulse of knee extension moments per indicator. "Cutting", which had the second largest angular impulse of knee extension moment at 1.0 Nm/kg or greater, was the largest at 1.5 Nm/kg or greater, followed by "Jump Single Leg Landing". Based on the number of motions with which statistically significant differences were observed, sharp changes in direction were the most load-intensive motion at 1.5 Nm/kg or greater, and single-legged landing after a jump was the second largest. Of the stop motions that were load-intensive for a moment of 1.0 Nm/kg or greater, "Approach Stop" was the third largest for the moment at 1.5 Nm/kg or greater, and significantly greater than 5 types of motion, suggesting that a sharp stop was a load-intensive motion. "Approach Stop & Back" was the fifth largest with a statistically significant difference only with both types of squats, thus a motion involving a stop followed by a backward run was deemed to be a load-intensive motion for a moment of 1.5 Nm/kg or greater.

"Cutting" had the largest angular impulse of the knee extension moment greater than or equal to 2.0 Nm/kg, which was significantly greater than 8 types of motion ranked below, and "Jump Single Leg Landing" was the second largest and significantly greater than 7 types of motion. Based on the number of types of motion with which statistically significant differences were observed, sharp changes in direction were the most load-intensive motion when the moment was 2.0 Nm/kg or greater, and single-legged landing after a jump was the second largest. At 1.0 Nm/kg or greater, stop motions were viewed as load-intensive motions, but they could not be considered as a large load when the moment was at 2.0 Nm/kg or greater.

"Cutting" had the largest angular impulse of "knee extension" moment also at 2.5 Nm/kg or greater, followed by "Jump Single Leg Landing". "Cutting" was significantly greater than the 7 types of motion ranked below, while "Jump Single Leg Landing" was significantly greater than the 5 types of motion ranked below. Based on the number of types of motion with which statistically significant differences were observed, sharp changes in direction had a greater load than single-legged landings after a jump. Since statistically significant differences were not observed for "Kick Flexion", ranked third, and other motions ranked below, a sharp change in direction and single-legged landings after a jump were deemed to be motions with a load greater than the other 12 types of motion.

Studies have suggested that Osgood–Schlatter disease is caused by an inflammation from repeated friction of the quadriceps on the epiphyseal cartilage in the tibial tubercle during a kinetically weak apophyseal stage [3–5]. Based on the peak value and angular impulse of the knee extension moment, this study showed that single-legged landing after a jump and a sharp change in direction (cutting) exert the largest load on the tibial tubercle among the 14 types of motion, and therefore possess high risk for onset and aggravation of Osgood–Schlatter disease. Stops were load-intensive and therefore a high risk for the development and aggravation of the disease, albeit to a lesser extent compared to single-legged landing after a jump or a sharp change in direction, but the results also suggested that the stop motion cannot be viewed as high-risk, depending on the indicator. Many studies have cited running as a motion that may cause Osgood–Schlatter disease, but this study identified motions that had a larger load than running and therefore cause a higher risk of developing and aggravation the disease. Loads from squats were small and their risk of causing the disease was low. However, specific values of the load that cause and aggravate Osgood–Schlatter disease is still unknown, and every motion assessed by this study may be a high risk or a low risk for developing the disease. This study also defined indicators as a moment of 1.0 Nm/kg or greater, 1.5 Nm/kg or greater, 2.0 Nm/kg or greater, and 2.5 Nm/kg or greater upon calculating the angular impulse of knee extension moment, but risk assessment varies by indicator in some motions such as stops. Motions with a high risk of developing and aggravating Osgood–Schlatter disease, as well as low risk motions, can be assessed in further detail based on this study if future studies can quantify the load and the number of repetitions that may cause and make worse Osgood–Schlatter disease while taking age and development stage into account.

Over 50% of Osgood–Schlatter disease patients have pain in both legs [29]. Both legs make similar movements in running, squats, and jumps (landing with both legs) while movements vary between the left and right legs in jumps (single-legged landing), changes in direction (cutting), stops, and turns. However, there was no need to analyze the load on the tibial tubercle of both legs since it was clear that the load on the right leg was smaller than the left, analyzed by this study. In kicking motions, however, the tibial tubercle in the kicking leg may also incur a large load due to the impact of the ball. This study identified the load on the tibial tubercle in the axial leg during a ball kicking motion, but the load on the kicking leg remains unknown. Hence, there is a need to analyze the load on the tibial tubercle in the kicking leg, including the position of impact.

If training regimens that balance the load on the tibial tubercle by scheduling a training day with low-load motions after a day that involved many load-intensive motions, the athletes could be protected from Osgood–Schlatter disease and increase their participation in sports. Motions analyzed by this study include those involved in many sports, including jumping and running. Therefore, Osgood–Schlatter disease may be prevented by preventing continuous load-intensive training sessions across various sports.

## Supporting information

S1 Fig Picture of the body with 18 markers. (TIF)

S1 Movie Motion of "Kick Normal". (MP4)

S2 Movie Motion of "Kick Flexion". (MP4)

S3 Movie Motion of "Kick Extension". (MP4)

S4 Movie Motion of "Kick Run". (MP4)

S5 Movie Motion of "Run Slow". (MP4)

S6 Movie Motion of "Run Fast". (MP4)

S7 Movie Motion of "Squat Hip". (MP4)

S8 Movie Motion of "Squat Knee". (MP4)

S9 Movie Motion of "Jump Both Legs Landing". (MP4)

S10 Movie Motion of "Jump Single Leg Landing". (MP4)

S11 Movie Motion of "Turn". (MP4)

S12 Movie Motion of "Cutting". (MP4)

S13 Movie Motion of "Approach Stop". (MP4)

S14 Movie Motion of "Approach Stop & Back". (MP4)
