# Supplementary figures and images for: Risk assessment of the onset of Osgood–Schlatter disease using kinetic analysis of various motions in sports
Source: PLoS One. 2018 Jan 8;13(1):e0190503. doi: 10.1371/journal.pone.0190503 (PMC5757930; doi:10.1371/journal.pone.0190503)

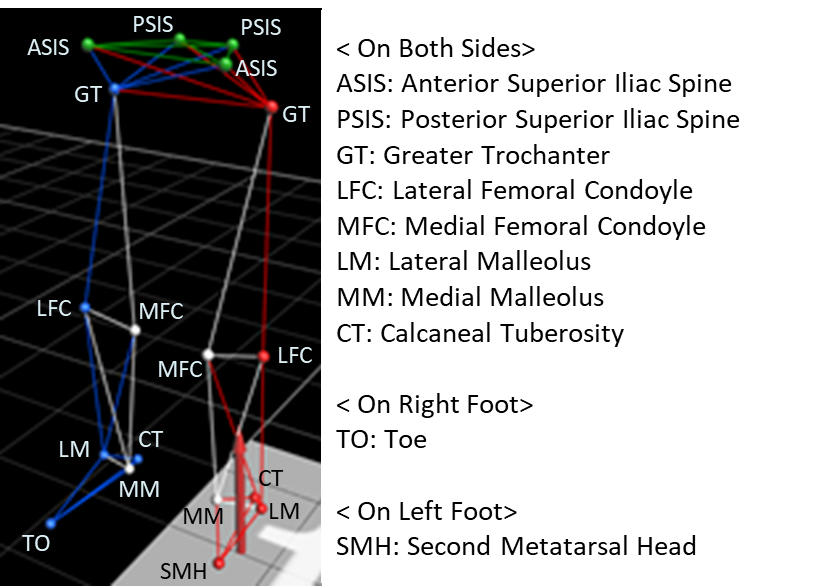

Supplement: S1 Fig — (TIF) [file pone.0190503.s001.tif]
